# GSDMD protects intestinal epithelial cells against bacterial infections through its N-terminal activity affecting intestinal immune homeostasis

**DOI:** 10.7555/JBR.38.20240041

**Published:** 2024-05-29

**Authors:** Honghui Li, Jie Pu, Dongxue Yang, Lu Liu, Yingchao Hu, Shuo Yang, Bingwei Wang

**Affiliations:** 1 Department of Immunology, State Key Laboratory of Reproductive Medicine, Jiangsu Key Lab of Cancer Biomarkers, Prevention and Treatment, Collaborative Innovation Center for Personalized Cancer Medicine, Gusu School, the Affiliated Wuxi People's Hospital of Nanjing Medical University, Wuxi People's Hospital, Wuxi Medical Center, Nanjing Medical University, Nanjing, Jiangsu 211166, China; 2 Department of Pharmacology, Nanjing University of Chinese Medicine, Nanjing, Jiangsu 210023, China

**Keywords:** GSDMD, pyroptosis, *Citrobacter rodentium* infection, intestinal mucosal barrier

## Abstract

The intestinal mucosal barrier serves as a vital guardian of the gut health, maintaining a delicate equilibrium between gut microbiota and host immune homeostasis. Gasdermin D (GSDMD), a key executioner of pyroptosis downstream of the inflammasome, has been found to play intricate roles in modulating colitis by influencing intestinal macrophages and regulating mucus secretion from goblet cells. However, the exact nature of the regulatory function of GSDMD in maintaining intestinal immune homeostasis and defending against pathogens remains to be elucidated. In the current study, by using the *Citrobacter rodentium* infection model, we found that GSDMD played a key role in the defense against intestinal *Citrobacter rodentium* infection, with high expression levels in intestinal epithelial and lamina propria myeloid cells. Our results showed that GSDMD acted specifically in intestinal epithelial cells to combat the infection, independently of its effects on antimicrobial peptides or mucin secretion. Instead, the resistance was mediated by the N-terminal fragment of GSDMD, highlighting its importance in intestinal immunity. However, the specific mechanism underlying the N-terminal activity of GSDMD in protecting against intestinal bacterial infections requires future investigation.

## Introduction

The intestinal mucosal barrier, consisting of biological, chemical, mechanical, and immune barriers, functions as a critical line of defense protecting the body from invading pathogens and is essential for maintaining the balance of gut microbiota and mucosal immune homeostasis ^[ 1– 2] ^. During intestinal bacterial infection, pattern recognition receptors on intestinal epithelial cells (IECs) recognize pathogen-associated molecular patterns and become activated, leading to the production of inflammatory factors and other immune defense molecules, thereby promoting the mucosal immune response ^[ 3] ^.

Inflammasomes are the key inflammatory protein machines of the innate immune system, with core components such as NOD-like receptors that sense various infections and endogenous danger signals, leading to the activation of inflammatory responses ^[ 4] ^. The inflammasome participates in host defense responses against pathogens and plays a crucial role in maintaining intestinal immune homeostasis ^[ 5– 6] ^.

Gasdermin D (GSDMD) is a key executioner of pyroptosis downstream of the inflammasome ^[ 7– 9] ^. Upon activation by the pathogen-associated molecular pattern signals through canonical or non-canonical pyroptosis pathways, the activated N-terminal fragment of GSDMD targets the cell membrane and oligomerizes to form pores, thereby exerting biological functions through pyroptosis or non-pyroptosis pathways ^[ 7, 10– 12] ^. GSDMD plays a critical role in numerous immunoinflammatory diseases, such as sepsis, neurodegenerative diseases, inflammatory bowel disease, atherosclerosis, and cancer ^[ 13– 23] ^.

Zhang *et al* ^[ 24]^ reported the role of GSDMD in the intestinal epithelium, where it regulated the mucus secretion from goblet cells to maintain the intestinal mucosal barrier. Additionally, our previous study found that GSDMD in intestinal macrophages controlled colitis by regulating cGAS-mediated inflammatory responses ^[ 25] ^. These findings underscore the complex and multifaceted nature of the regulatory functions of GSDMD in maintaining intestinal immune homeostasis and defending against pathogens. However, the exact role of GSDMD in pathogen defense remains to be elucidated.

In the current study, we used a *Citrobacter rodentium* ( *C. rodentium*) infection model to identify the role that GSDMD might play against intestinal bacterial infections exclusively in intestinal epithelial cells, and whether this role depended on its N-terminal active fragment, the expression of antimicrobial peptides, or the secretion of mucus proteins.

## Materials and methods

### Mice

Male mice with the C57BL/6 background were used in the current study. The *Gsdmd* ^−/−^ mice were kindly provided by F. Shao (National Institute of Biological Sciences, China). The *Villin*-CreERT mice were kindly provided by Dr. Yeguang Chen (Tsinghua University, China). The *Asc* ^−/−^ mice were a gift from V. Dixit (Genentech). The *Gsdmd* ^fl/fl^ and the *Gsdmd*-NT ^F/+^mice were generated using conditional gene targeting methods by Cyagen Biosciences Inc. (Guangzhou, China), as described previously ^[ 12, 21] ^. For the *Gsdmd*-NT ^F/+^ mice, the donor DNA containing the 3×Flag *Gsdmd*-N fragment with LoxP-flanked stop cassette, the gRNA targeting the mouse *Rosa26* gene, and *Cas9* mRNA were co-injected into the C57BL/6 zygotes to generate the targeted knock-in offspring. *Gsdmd*-floxed mice were crossed with lysozyme M-Cre mice ( *Lysm*-Cre; Jackson Laboratory) to generate myeloid cell-conditional *Gsdmd* knockout mice ( *Gsdmd* ^fl/fl^ *Lysm*-Cre) or with *Villin*-Cre mice (Jackson Laboratory) to produce IEC-conditional *Gsdmd* knockout mice ( *Gsdmd* ^fl/fl^ *Villin*-Cre). *Gsdmd*-NT ^F/+^mice were crossed with *Villin*-CreERT mice to produce IEC-conditional *Gsdmd* knockin mice ( *Gsdmd*-NT ^F/+^ *Villin*-CreERT). *Gsdmd*-NT ^F/+^ *Villin*-CreERT mice were crossed with *Gsdmd* ^−/−^ mice to produce IEC-conditional *Gsdmd* rescue mice. To induce the activity of Cre, male mice aged eight to ten weeks were injected intraperitoneally with 300 μL tamoxifen (10 mg/mL) five days before the establishment of the *C. rodentium* infection model. All mice were kept in a barrier facility, and all animal experiments were conducted according to the procedures approved by the Ethical Review Committee for Laboratory Animal Welfare of Nanjing Medical University and Nanjing University of Chinese Medicine.

### Reagents and materials

*C. rodentium* (Cat. #51459, strain ICC180) was obtained from ATCC (Manassas, VA, USA). Potassium salt was purchased from Sciencelight (Cat. #luc001, Shanghai, China). Tris, Igepal, glycerol, NaF, Na _3_VO _4_, dithiothreitol, NaCl, anhydrous ethanol, xylene, neutral gum, hydrochloric acid, and paraformaldehyde were sourced from Sinopharm Chemical Reagent Co., Ltd. (Shanghai, China). Agarose was supplied by Yeasen (Cat. #10208ES60, Shanghai, China). Yeast extract (LP0021) and Tryptone (LP0042) were provided by OXOID (Waltham, MA, USA). Phenylmethylsulphonyl fluoride (Cat. #52332), Nalidixic acid (Cat. #N8878), Hematoxylin (Cat. #HHS32), Eosin (Cat. #E4382), and protease inhibitor cocktail (Cat. #P8340) were obtained from Sigma-Aldrich (City of Saint Louis, MO, USA). Streptomycin sulfate was sourced from Ameresco (Cat. #S0382, Framingham, MA, USA). TRIzol reagent was purchased from Invitrogen (Cat. #15596026, Carlsbad, CA, USA). AceQ qPCR SYBR Green Master Mix was purchased from Vazyme (Cat. #Q131-02/03, Nanjing, China). Gentamycin was purchased from Sangon Biotech (Cat. #B540724-0010, Shanghai, China). Kanamycin sulfate was obtained from Biosharp (Cat. #0408, Anhui, China). Anti-interleukin (IL)-6 (Cat. #DY406), anti-tumor necrosis factor (TNF)-α (Cat. #DY410), and anti-monocyte chemotactic protein (MCP-1; Cat. #DY479) enzyme-linked immunosorbent assay kits were provided by R&D Systems (Minneapolis, MN, USA). Periodic acid-Schiff (PAS) staining solution was sourced from Wuhan Guge Biological Technology Co., Ltd. (Wuhan, China). Anti-EPCAM (Cat. #567664, 1 : 200) and CX3CR1 (Cat. #567805, 1 : 500) were purchased from BD Biosciences (Sussex, NJ, USA). Anti-CD45-AF700 (Cat. #56-0451-82, 1 : 400) and anti-CD326-APC (Cat. #17-5791-80, 1 : 400) were purchased from eBioscience (Santiago, CA, USA).

### *C. rodentium* bacterial culture

The *C. rodentium* strain was propagated overnight on agar plates, then a single clone was isolated and inoculated in lysogeny broth agar medium (containing kanamycin sulfate and nalidixic acid) until it grew to an exponential phase (OD600 = 0.5−1.0). The cells were centrifuged at 12000 *g* for 10 min at 4 ℃, and the pellets were washed once with pre-cooled phosphate-buffered saline (PBS) and then stored in PBS.

### Optical imaging *in vivo*

Eight to ten-week-old mice were deprived of food and water for 4 h before being gavaged with 25 mg of streptomycin sulfate. After 24 h, the mice were deprived of food and water for another 4 h before being gavaged with bioluminescent *C. rodentium* (ICC180) (2 × 10 ^9^ CFU). The mice were then given a normal diet 4 h later to establish the enteritis model. On the 9th day post intragastric administration, the mice were subjected to optical imaging *in vivo*, using the IVIS Spectrum instrument (Caliper, PerkinElmer) to collect images and Living Images 4.3.1 software for data analysis.

### Bacterial load test

On the 15th day after intragastric administration of *C. rodentium* (ICC180), the mouse organs (mesenteric lymph nodes, spleen, and liver) were collected, weighed, and then added to sterile PBS and ground with ceramic beads under sterile conditions. After grinding, the cells were centrifuged at 1000 *g*, and plating was started. After the plating was completed, the culture dish was inverted at 37 ℃ overnight to observe the bacterial growth and perform statistical analysis.

### Hematoxylin and eosin (H&E) staining

Mouse colon tissues were fixed in a 4% paraformaldehyde solution for more than 24 h. The tissues were dehydrated, cleared, and immersed in wax before being embedded in paraffin and sectioned. After routine dewaxing with xylene and rehydration using a gradient of alcohol from high to low, the sections were rinsed with tap water for 5 min, followed by a hematoxylin staining for 3 min. They were then rinsed with tap water for 5 min, differentiated with 1% hydrochloric acid alcohol for 3–5 s, rinsed with running water for 5–10 min, blued with ammonia, and rinsed with tap water for 5 min. The sections were further stained with eosin solution for 15 min, rinsed with tap water for 2 min, dehydrated with a gradient of alcohol from low to high, cleared with xylene, and sealed with neutral gum. Images were captured using a Nikon 50i microscope, followed by analyzing the degree of intestinal inflammatory cell infiltration and mucosal hyperplasia.

### Immunofluorescence staining

Tissue sections were incubated at 4 ℃ overnight with primary antibodies to GSDMD, EPCAM, and CX3CR1. The slides were then incubated with the indicated secondary antibodies. The nuclei were counterstained with 4′,6-diamidino-2-phenylindole (DAPI) (Sigma-Aldrich, SL, USA). After drying, the slides were mounted using ProLong Antifade Mounting Medium (Beyotime Biotechnology, Shanghai, China), and visualized using either a Nikon 50i fluorescence microscope or a Zeiss LSM 700 META laser scanning confocal microscope.

### PAS staining

Tissue processing for paraffin-embedded sections was performed as previously described in the H&E staining. The sections were dipped in PAS staining solution in the dark for 45–60 min, rinsed with tap water for 10 min, and then dipped in hematoxylin staining solution for 3 min. After rinsing with tap water for 5 min, the sections were differentiated with hydrochloric acid alcohol solution for 3–5 s, rinsed again with tap water for 5–10 min, and blued with ammonia. The sections were further rinsed with tap water for 5 min, dehydrated with a gradient of alcohol from low to high, cleared with xylene, and sealed with neutral gum. Images were captured using a Nikon 50i microscope and analyzed.

### Real-time reverse transcription-PCR (RT-qPCR)

The colon tissues were collected and centrifuged. Total RNA was extracted using TRIzol reagent (Invitrogen, USA) and subjected to cDNA synthesis. qRT-PCR was performed using AceQ qPCR SYBR Green Master Mix (Vazyme, China). The expression of a single gene was calculated using a standard curve method and standardized to the expression of *Hprt*. The following primers were used: mouse *Dfn5*, forward, 5ʹ-GGCTGATCCTATCCACAAAACA-3ʹ, reverse, 5ʹ-AGACCCTTCTTGGCCTCCA-3ʹ; mouse *Il22*, forward, 5ʹ-CCCTTATGGGGACTTTGGC-3ʹ, reverse, 5ʹ-GGTGCGGTTGACGATGTATG-3ʹ; mouse *Reg3g*, forward, 5ʹ-AACAGAGGTGGATGGGAGTGG-3ʹ, reverse, 5ʹ-CACAGTGATTGCCTGAGGAAGA-3ʹ; mouse *Muc2*, forward, 5ʹ-TTGCTCTGCTGTCTCCGTCA-3ʹ, reverse, 5ʹ-ACACTGGTCTTCTCCTCCTTGC-3ʹ; mouse *Reg3b*, forward, 5ʹ-AATGGAGGTGGATGGGAATG-3ʹ, reverse, 5ʹ-CGGTCTAAGGCAGTAGATGGGT-3ʹ; mouse *Lysozyme1*, forward, 5ʹ-ACTCTGGGACTCCTCCTGCTT-3ʹ, reverse, 5ʹ-CGGTCTCCACGGTTGTAGTTT-3ʹ; mouse *Villin*, forward, 5ʹ-CAGAATGGTGGACGATGGCTCT-3ʹ, reverse, 5ʹ-GACAAGGTAGCAGTTTCCTGAGC-3ʹ; mouse *Cd45*, forward, 5ʹ-CTTCAGTGGTCCCATTGTGGTG-3ʹ, reverse, 5ʹ-TCAGACACCTCTGTCGCCTTAG-3ʹ; mouse *Hprt*, forward, 5ʹ-GTCCCAGCGTCGTGATTAGC-3ʹ, reverse, 5ʹ-TGGCCTCCCATCTCCTTCA-3ʹ.

### Enzyme-linked immunosorbent assay (ELISA)

On the 15th day after intragastric administration of *C.rodentium* (ICC180), the colon tissues were weighed and homogenized. After grinding, the cells were centrifuged at 12500 *g* at 4 ℃. Conditioned media were collected and analyzed for the levels of IL-6, TNF-α, and MCP-1, according to the manufacturer's instructions.

### Western blotting

The colon tissues or bone marrow-derived macrophage (BMDM) cells were collected in lysis buffer (50 mmol/L Tris-HCl, pH 7.4, containing 150 mmol/L NaCl, 1% Igepal, 10% glycerol, 50 mmol/L NaF, 1 mmol/L Na _3_VO _4_, 1 mmol/L dithiothreitol, 1 mmol/L phenylmethylsulphonyl fluoride, and complete protease inhibitor cocktail), and incubated for 40 min at 4 ℃. Samples were quantified and resolved by SDS-PAGE, then transferred to nitrocellulose membranes. The membranes were immunoblotted with primary antibodies, and proteins were detected using an appropriate secondary anti-rabbit antibody conjugated to fluorescence. Immunoreactivity was visualized by the Odyssey Imaging System (LI-COR Biosciences, Lincoln, NE, USA).

### Isolation of colonic epithelial and immune cells and sorting

Mouse colons were excised and thoroughly washed with PBS several times. They were opened longitudinally and transferred into Gentle Cell Dissociation Reagent (Cat. #50-239-0198, STEMCELL, Vancouver, BC, Canada) and shaken at 37 ℃ for 20 min. The colons were then washed three times with PBS containing 2 mmol/L EDTA ^[ 26] ^. Supernatants were collected and passed through a 100 µm cell strainer to obtain single-cell suspensions. The single-cell suspensions were collected and stained with anti-CD45 and anti-CD326. IECs (CD326 ^+^CD45 ^−^) were sorted on a BD FACSAria. The remaining colons were collected and digested at 37 ℃ for 45 min using Dulbecco's Modified Eagle Medium containing 2% fetal bovine serum, collagenase Ⅳ (2.5 mg/mL; Cat. #9001-12-1, Sigma-Aldrich), and deoxyribonuclease Ⅰ (10 U/mL; Cat. #10104159001, Roche, MA, USA). Single-cell suspensions were obtained by grinding through a 70 µm cell strainer. Subsequently, homogeneous cell suspensions were centrifuged over the Percoll density (Cat. #17-0891-02, GE Healthcare, USA), and lamina propria immune cells were separated by collecting the interface fractions between 40% and 80% Percoll. After intensive washing, single-cell suspensions were stained with FVD eFlour 506, anti-CD45, and anti-CD326 for the fluorescence-activated cell sorting.

### Statistical analysis

Data were presented as the mean ± standard error of the mean. Samples were analyzed using an unpaired Student's *t*-test or Mann-Whitney test for two groups and ANOVA for multiple groups. In all cases, a *P*-value of less than 0.05 was considered statistically significant.

## Results

### Protective role of GSDMD against the colon *C. rodentium* infection

The *C. rodentium* infection model in mice is commonly used to simulate human enteropathogenic *Escherichia coli* ( *E. coli*) infection, inducing inflammatory and pathological responses similar to those caused by human enteropathogenic and enterohemorrhagic *E. coli*. To investigate the role of GSDMD in host defense against intestinal bacterial infections, we used *C. rodentium* to infect the *Gsdmd* knockout and wildtype mice. One previous study demonstrated that the deletion of *Asc* (apoptosis-associated speck-like protein containing a CARD) aggravated the *C. rodentium* infection in the colon of mice by promoting bacterial colonization ^[ 27] ^, rendering the *Asc* ^−/−^ mice a valuable tool for comparing the effects of GSDMD deficiency on infection outcomes. Consequently, the *Asc* knockout mice were used as controls in the current study.

**Figure 1:**
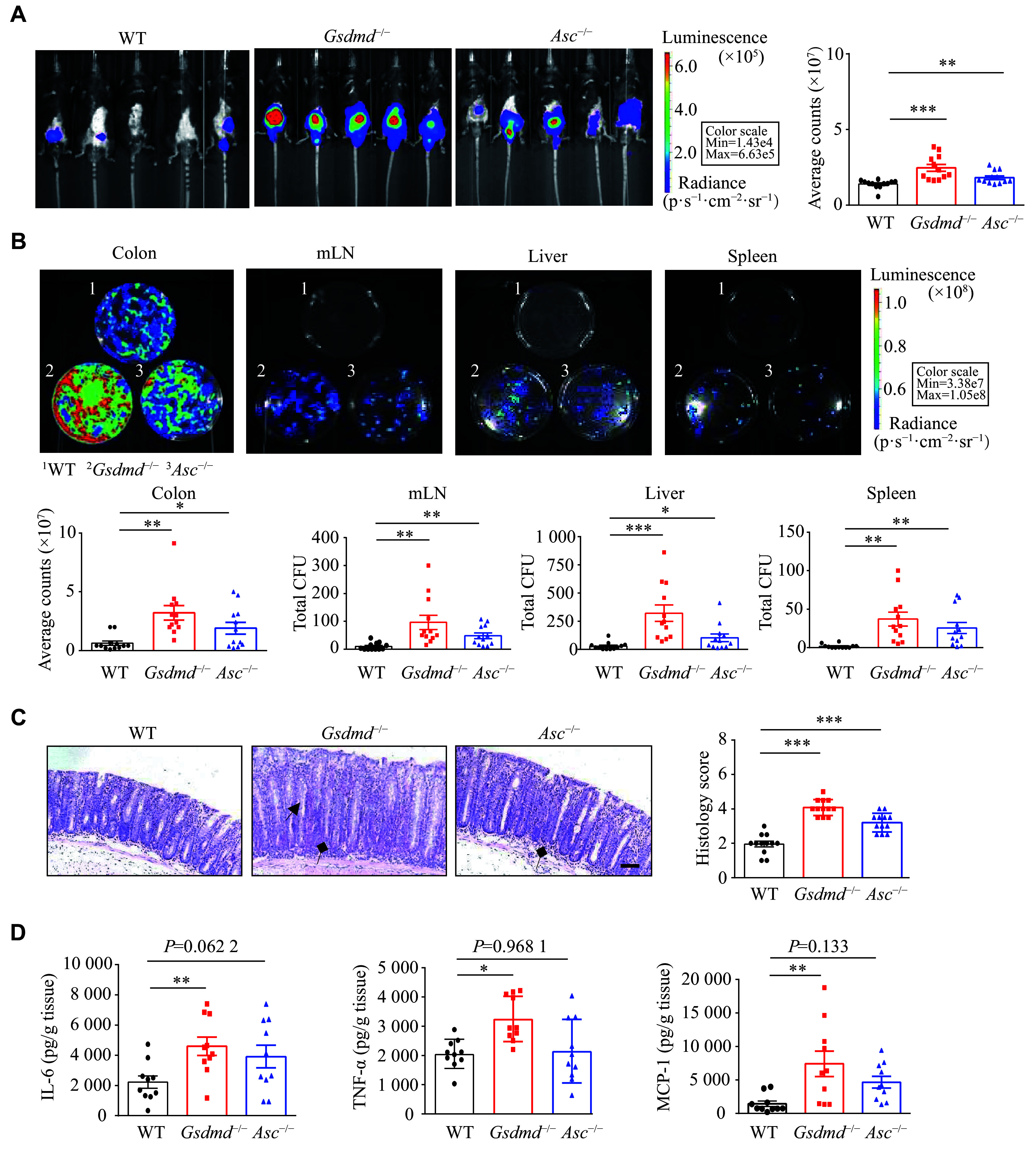
GSDMD defended against the *C. rodentium* infection in the colon. A: Optical imaging *in vivo* analysis of the bacterial growth of WT, *Gsdmd* ^−/−^and *Asc* ^−/−^mice on the 9th day after intragastric administration of *C. rodentium*. Data are presented as a representative image (left) and quantified intensity (right) ( *n* = 12). B: Counts of bacterial colonies in the colon, mesenteric lymph nodes (mLN), liver, and spleen of the WT, *Gsdmd* ^−/−^and *Asc* ^−/−^mice on the 15th day after intragastric administration of *C. rodentium* ( *n* = 12). C: Representative images and histological scores of the H&E staining showing colon pathological injury of the WT, *Gsdmd* ^−/−^and *Asc* ^−/−^mice on the 15th day after intragastric administration of *C. rodentium*. Scale bars, 200 μm ( *n* = 12). The triangular arrows and square arrows indicate intestinal tissue disorganization and immune cell infiltration, respectively. D: The ELISA analysis of IL-6, TNF-α, and MCP-1 in the colon supernatant of the WT, *Gsdmd* ^−/−^and *Asc* ^−/−^mice on the 15th day after intragastric administration of *C. rodentium* ( *n* = 10). Data are pooled from three independent experiments (A–D). ^*^ *P* < 0.05, ^**^ *P* < 0.01, and ^***^ *P* < 0.001 by unpaired Student's *t*-test (A–D).

We observed that the *Gsdmd* ^−/−^ mice had a phenotype consistent with the *Asc* ^−/−^ mice, exhibiting stronger and more widespread *C. rodentium* bioluminescence signals in the body at day 9 post-infection, compared with the WT mice ( ***Fig. 1A***). On day 15 post-infection, the *Gsdmd* ^−/−^ mice exhibited an increased bacterial burden in the colon, liver, mesenteric lymph nodes, and spleen ( ***Fig. 1B***), as well as the thickened colon mucosa and increased immune cell infiltration ( ***Fig. 1C***), accompanied by elevated levels of the pro-inflammatory cytokines like IL-6, TNF-α, and MCP-1 ( ***Fig. 1D***), compared with the WT mice. These findings suggest that GSDMD may play a crucial role in resisting the *C. rodentium* infection in the colon.

### High expression of GSDMD in colon epithelial and lamina propria myeloid cells

**Figure 2:**
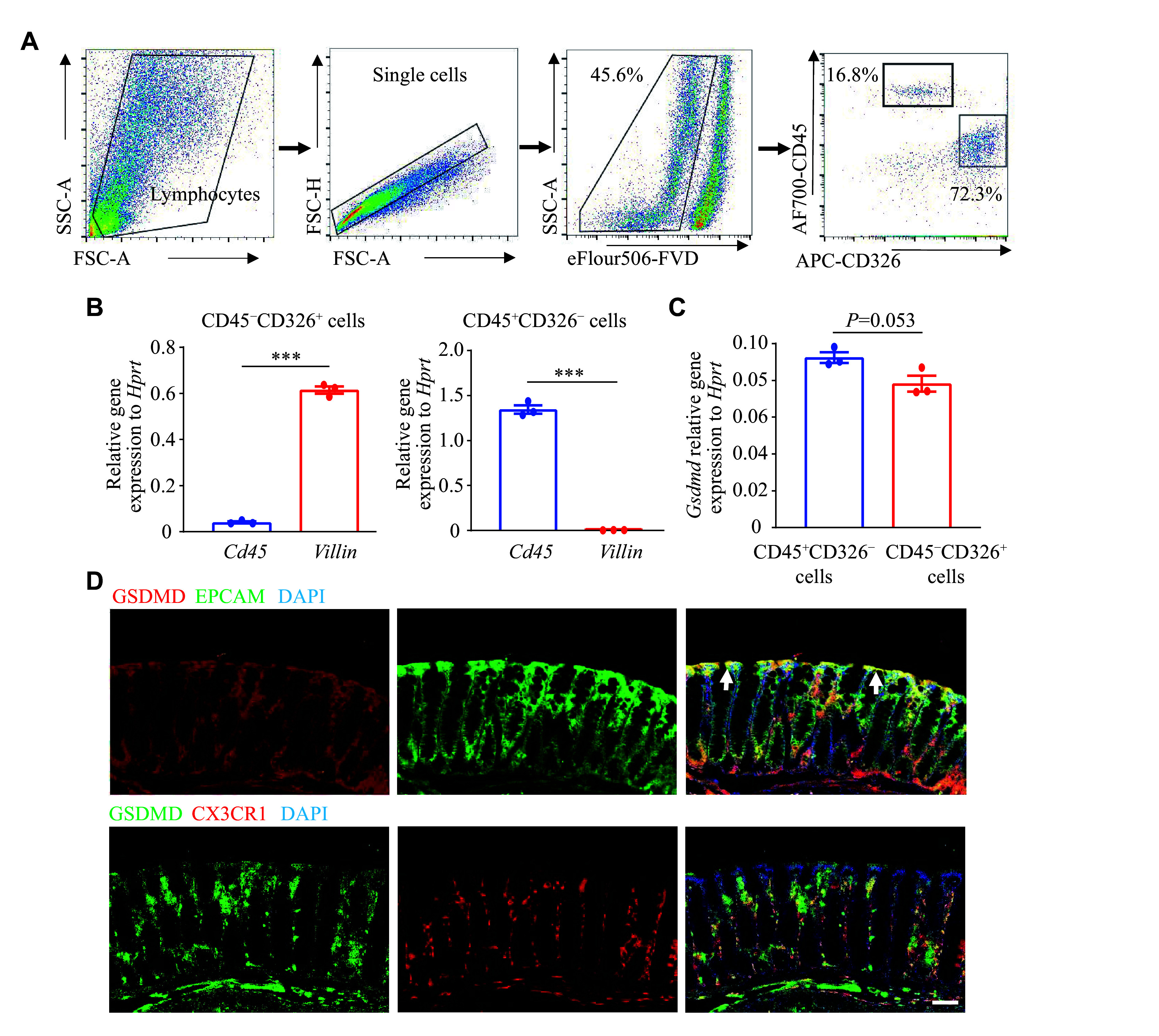
GSDMD exhibited high expression in colon epithelial and lamina propria myeloid cells. A–C: The real-time transcription-PCR analysis of *Gsdmd* expression in sorted colon epithelial cells and myeloid cells by flow cytometry ( *n* = 3). D: Immunofluorescence analysis of the distribution of GSDMD in colon epithelial cells and myeloid cells. Scale bars, 200 μm. The arrows indicate the merging of GSDMD with EPCAM. Data are pooled from three independent experiments (A–C) or are representative of three independent experiments (D). ^***^ *P* < 0.001 by unpaired Student's *t*-test (B and C).

To investigate the protective role of GSDMD in the defense against *C. rodentium*, we used flow cytometry sorting to obtain colon epithelial cells (CD326 ^+^CD45 ^−^) and hematopoietic cells (CD45 ^+^CD326 ^−^) ( ***Fig. 2A***). We then verified the expression levels of *Villin*, representing epithelial cells, and *Cd45*, representing myeloid cells, in these two cell populations using the RT-qPCR analysis. The results demonstrated the accuracy of flow cytometry sorting ( ***Fig. 2B***). We also examined the expression and localization of GSDMD in the colon and observed notably high expression levels of GSDMD in both colon epithelial cells and hematopoietic cells, which are critical components of the intestinal mucosal immune system ( ***Fig. 2C***). The results of immunofluorescence staining, using EPCAM and CX3CR1 as markers for epithelial and hematopoietic cells, respectively, further demonstrated the distribution of GSDMD in both colon epithelial cells and lamina propria hematopoietic cells ( ***Fig. 2D***).

### Functional defense of GSDMD against *C. rodentium* infection specifically in colon epithelial cells

**Figure 3:**
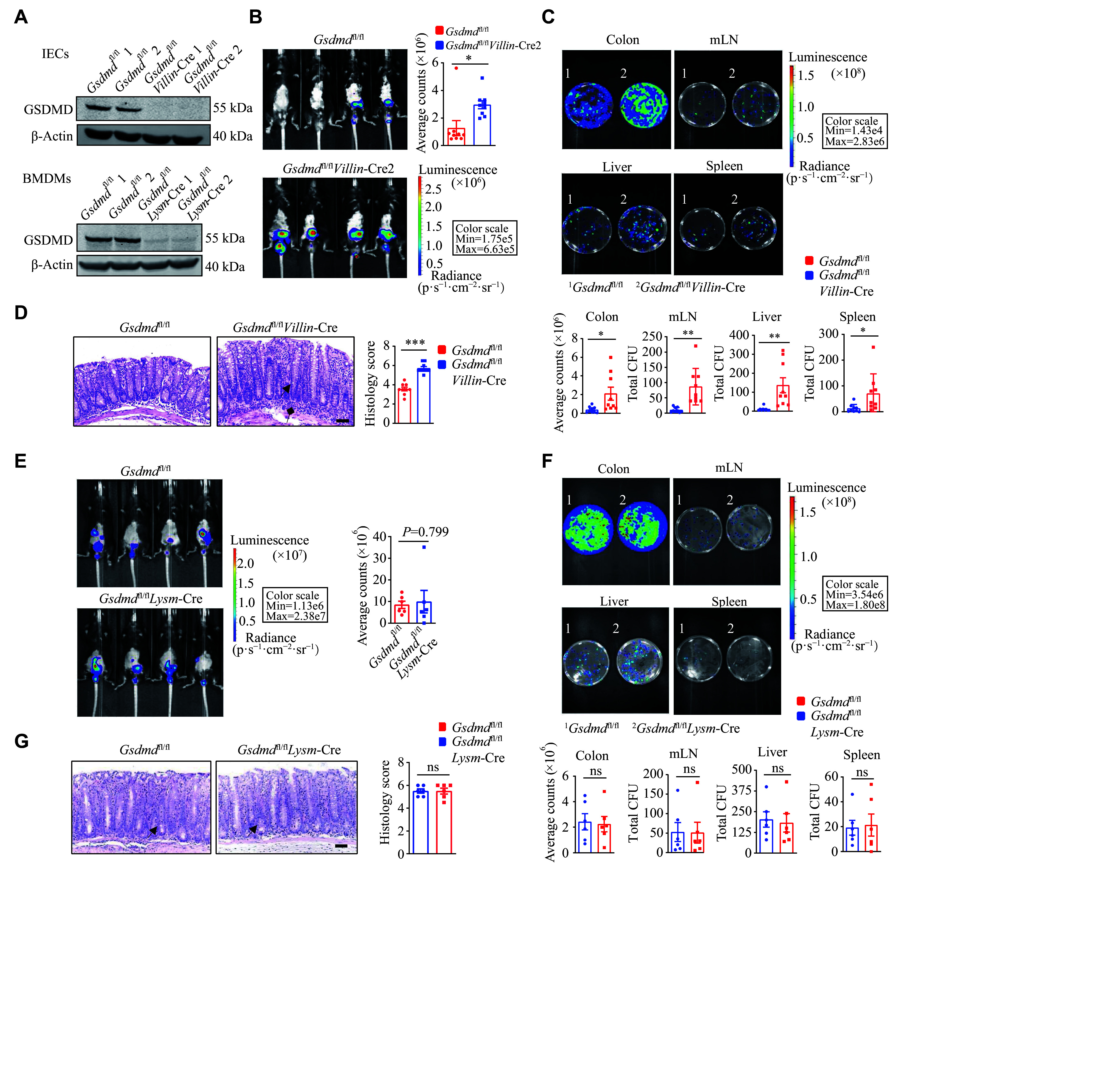
GSDMD functions in defense against the *C. rodentium* infection in colon epithelial cells. A: Immunoblot analysis of the knockout efficiency of GSDMD in colon epithelial cells and myeloid cells. B: Optical imaging *in vivo* analysis of the bacterial growth of the *Gsdmd* ^fl/fl^ and *Gsdmd* ^fl/fl^ *Villin*-Cre mice on the 9th day after intragastric administration of *C. rodentium*. Data are presented as a representative image (left) and quantified intensity (right) ( *n* = 9). C: Counts of bacterial colonies in the colon, mesenteric lymph nodes (mLN), liver, and spleen of the *Gsdmd* ^fl/fl^ and *Gsdmd* ^fl/fl^ *Villin*-Cre mice on the 15th day after intragastric administration of *C. rodentium* ( *n* = 9). D: Representative images and histological scores of H&E staining of colon pathological injury of the *Gsdmd* ^fl/fl^ and *Gsdmd* ^fl/fl^ *Villin*-Cre mice on the 15th day after intragastric administration of *C. rodentium*. Scale bars, 200 μm ( *n* = 12). The triangular arrows and square arrows indicate intestinal tissue disorganization and immune cell infiltration, respectively. E: Optical imaging *in vivo* analysis of the bacterial growth of the *Gsdmd* ^fl/fl^ and *Gsdmd* ^fl/fl^ *Lysm*-Cre mice on the 9th day after intragastric administration of *C. rodentium*. Data are presented as a representative image (left) and quantified intensity (right) ( *n* = 6). F: Counts of bacterial colonies in the colon, mesenteric lymph nodes, liver, and spleen of the *Gsdmd* ^fl/fl^ and *Gsdmd* ^fl/fl^ *Lysm*-Cre mice on the 15th day after intragastric administration of *C. rodentium* ( *n* = 6). G: Representative images and histological scores of H&E staining of colon pathological injury of the *Gsdmd* ^fl/fl^ and *Gsdmd* ^fl/fl^ *Lysm*-Cre mice on the 15th day after intragastric administration of *C. rodentium*. Scale bars, 200 μm ( *n* = 6). The triangular arrows indicate intestinal tissue disorganization. Data are representative of three independent experiments (A) or are pooled from three independent experiments (B–G). ^*^ *P* < 0.05, ^**^ *P* < 0.01, and ^***^ *P* < 0.001 by unpaired Student's *t*-test (B–G). Abbreviation: ns, not significant.

GSDMD is primarily expressed in myeloid cells ^[ 24] ^. Therefore, when *Gsdmd* was knocked out in myeloid cells using *Lysm*-Cre mice, there was essentially no GSDMD expression in the immune cells of these mice. To further elucidate the specific intestinal cells involved in the defense of GSDMD against bacterial infection, we performed *C. rodentium* infection studies in conditional *Gsdmd* knockout mice in intestinal epithelial ( *Villin*-Cre) and myeloid ( *Lysm*-Cre) cells ( ***Fig. 3A***). *In vivo* imaging results showed that the *Gsdmd* ^fl/fl^ *Villin*-Cre mice exhibited stronger and more widely distributed *C. rodentium* bioluminescent signals on the 9th day post-infection ( ***Fig. 3B***), compared with the controls. On the 15th day post-infection, *Gsdmd* ^fl/fl^ *Villin*-Cre mice demonstrated an increased bacterial burden in the colon, liver, mesenteric lymph nodes, and spleen ( ***Fig. 3C***), along with the thickened colon mucosa and enhanced immune cell infiltration ( ***Fig. 3D***). In contrast, on the 9th day post-infection, the *Gsdmd* ^fl/fl^ *Lysm*-Cre mice showed no significant difference in *C. rodentium* bioluminescent signals, compared with the controls ( ***Fig. 3E***). Moreover, there was no significant difference between the two groups in the colony burden of each tissue ( ***Fig. 3F***) or the colonic pathological score in mice on day 15 post-infection ( ***Fig. 3G***). Taken together, these findings indicate a critical protective role of GSDMD, specifically in colon epithelial cells, against intestinal bacterial infections.

### Lack of effect of GSDMD on antimicrobial peptide expression and mucin secretion in colon epithelial cells

**Figure 4:**
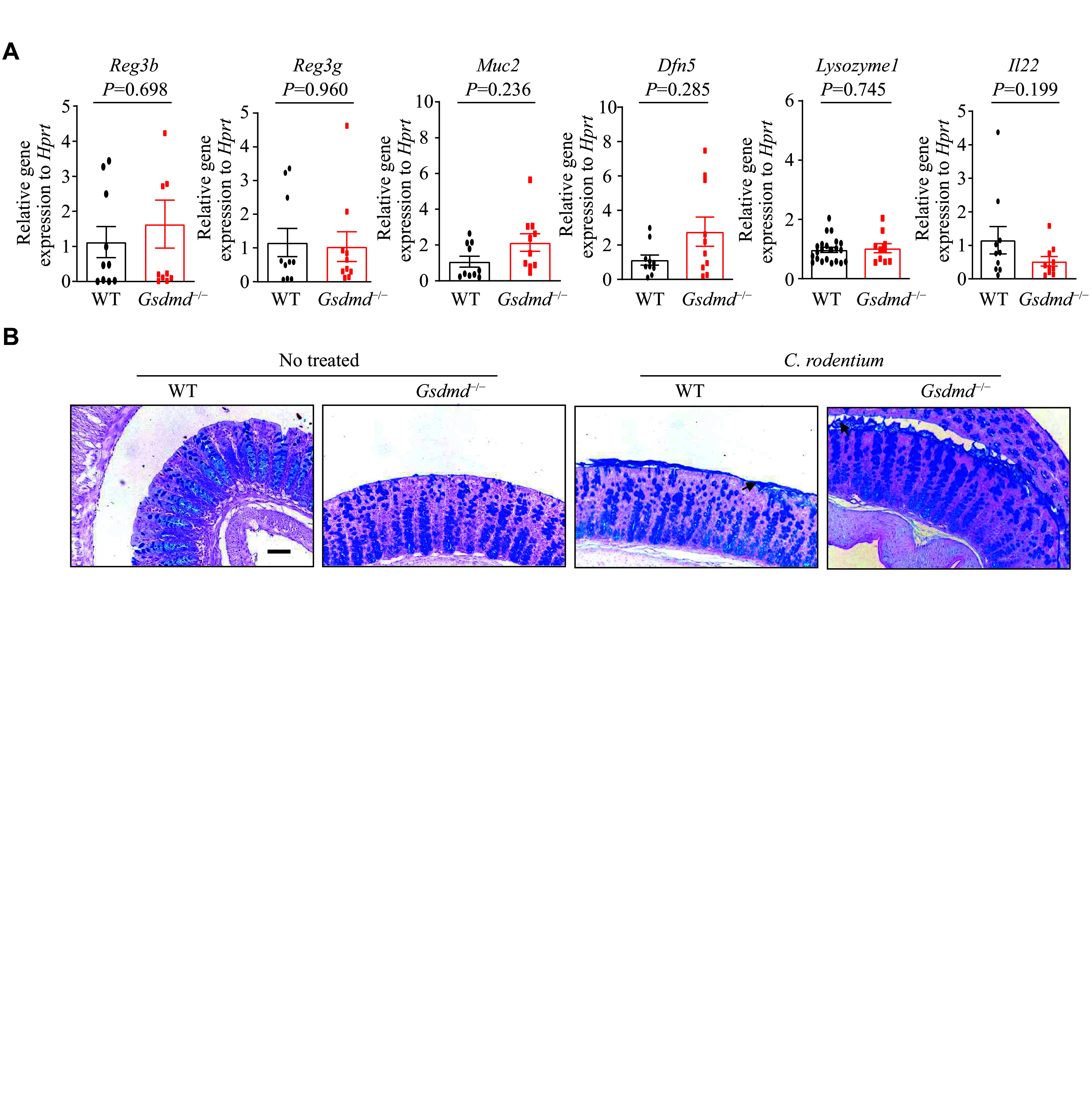
GSDMD did not affect the expression of antimicrobial peptides and mucin secretion in colon epithelial cells. A: The real-time transcription-PCR analysis of the expression of colon antimicrobial peptides of the WT and *Gsdmd* ^−/−^ mice ( *n* = 9). B: The periodic acid-Schiff staining analysis of the secretion of colon microbiota of the WT and *Gsdmd* ^−/−^ mice. Scale bars, 200 μm. Data are pooled from three independent experiments (A) or are representative of three independent experiments (B). The arrow indicates the secretion of mucus by the goblet cells in the colon tissue. *P*-values were determined by unpaired Student's *t*-test (A).

To gain insights into the mechanisms by which GSDMD defends against bacterial infections, we first evaluated the expression of antimicrobial peptides and mucosal-related proteins within the intestinal mucosa upon the *C. rodentium* infection. RT-qPCR analysis was performed to assess the transcriptional expression of genes vital for colon epithelium function and immunity, namely *Reg3 beta*, *Reg3 gamma*, *Muc2*, *Dfn5*, *Lysozyme1*, and *Il22*. The results showed that the GSDMD deletion did not significantly affect the expression levels of these genes in the colon epithelium ( ***Fig. 4A***). These findings indicate that while GSDMD may play a role in intestinal immunity and epithelium function, its absence does not alter the baseline transcriptional expression of these critical genes.

Given the regulatory function of GSDMD in the mucus secretion of goblet cells ^[ 24] ^, and the crucial role of intestinal mucin in the formation of the intestinal epithelial barrier, which separates the epithelium from intestinal flora and wards off invading pathogens ^[ 28] ^, we next investigated the secretion of colon mucins in response to the *C. rodentium* infection. The results of PAS staining, a method used to detect mucin, demonstrated that the secretion of mucins by goblet cells remained unaffected by the absence of GSDMD ( ***Fig. 4B***). These data indicate that GSDMD does not defend against bacterial infection by affecting the expression of antimicrobial peptides or the secretion of mucus proteins.

### GSDMD N-terminal fragments mediated resistance to the *C. rodentium* infection in colon epithelial cells

**Figure 5:**
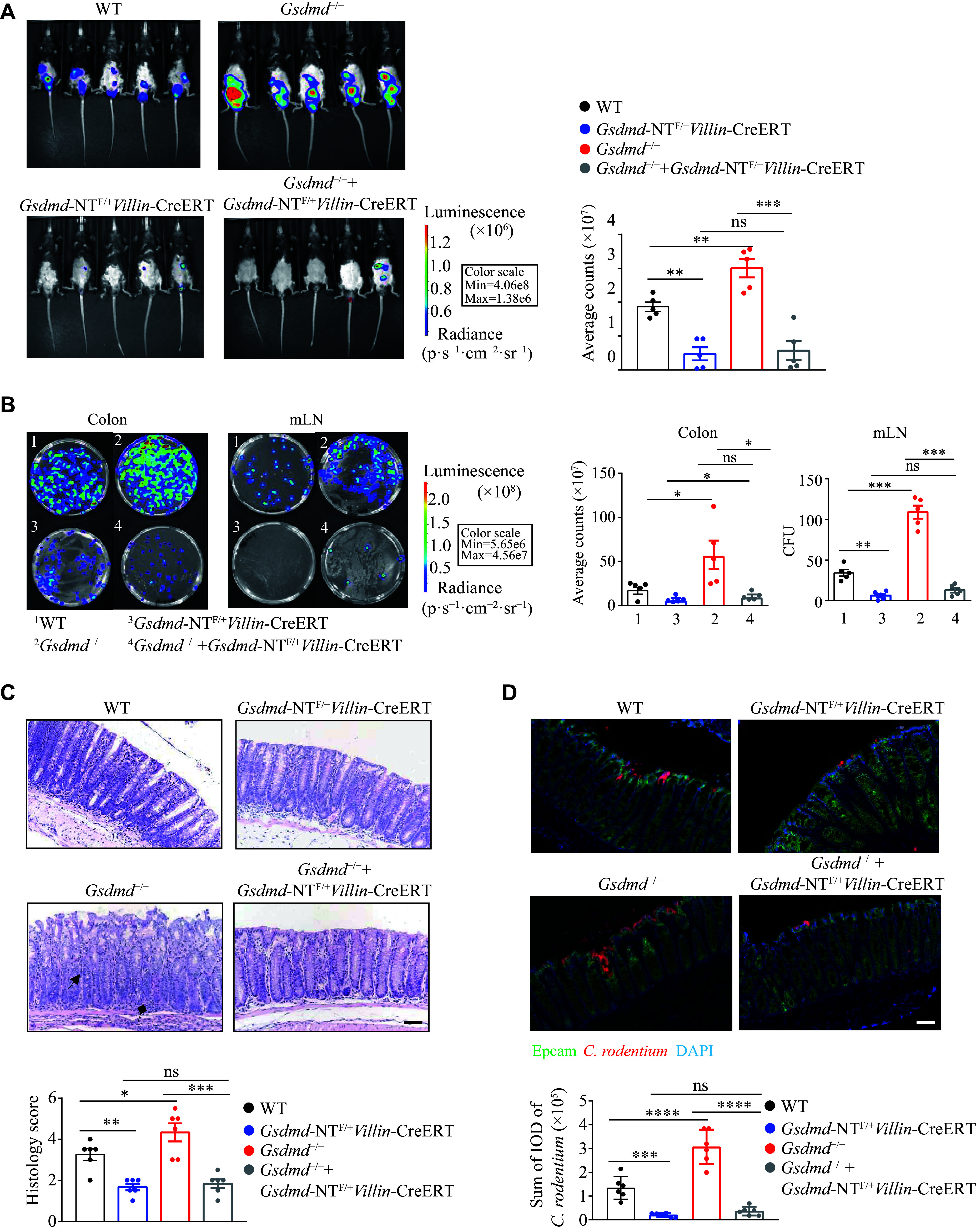
GSDMD resisted *C. rodentium* infection through N-terminal fragments in colon epithelial cells. A: Optical imaging *in vivo* analysis of the bacterial growth of WT, *Gsdmd* ^−/−^, *Gsdmd*-NT ^F/+^ *Villin*-CreERT, and *Gsdmd* ^−/−^ + *Gsdmd*-NT ^F/+^ *Villin*-CreERT mice on the 9th day after intragastric administration of *C. rodentium*. Data are presented as a representative image (left) and quantified intensity (right) ( *n* = 5). B: Counts of bacterial colonies in the colon and mesenteric lymph nodes (mLN) of WT, *Gsdmd* ^−/−^, *Gsdmd*-NT ^F/+^ *Villin*-CreERT, and *Gsdmd* ^−/−^ + *Gsdmd*-NT ^F/+^ *Villin*-CreERT mice on the 15th day after intragastric administration of *C. rodentium* ( *n* = 5). C: Representative images and histological scores of H&E staining of colon pathological injury of WT, *Gsdmd* ^−/−^, *Gsdmd*-NT ^F/+^ *Villin*-CreERT, and *Gsdmd* ^−/−^ + *Gsdmd*-NT ^F/+^ *Villin*-CreERT mice on the 15th day after intragastric administration of *C. rodentium*. Scale bars, 200 μm ( *n* = 6). The triangular arrows and square arrows indicate intestinal tissue disorganization and immune cell infiltration, respectively. D: Immunofluorescence analysis of the colonic bacterial colonization of WT, *Gsdmd* ^−/−^, *Gsdmd*-NT ^F/+^ *Villin*-CreERT, and *Gsdmd* ^−/−^ + *Gsdmd*-NT ^F/+^ *Villin*-CreERT mice on the 15th day after intragastric administration of *C. rodentium*. Scale bars, 200 μm ( *n* = 6). Data are pooled from three independent experiments (A–D). ^*^ *P* < 0.05, ^**^ *P* < 0.01, ^***^ *P* <0.001, and ^****^ *P* <0.0001 by unpaired Student's *t*-test (A–D). Abbreviation: ns, not significant.

Studies have reported that GSDMD undergoes cleavage at the Asp275 site to form a 22 kDa C-terminal fragment and a 31 kDa N-terminal fragment. The N-terminal fragment targets the plasma membrane and oligomerizes to form pores, thereby exerting its biological functions through pyroptosis or non-pyroptotic pathways ^[ 24, 29] ^. Consequently, we investigated the role of the GSDMD N-terminal fragment in colon epithelial cells in defense against pathogenic infections. We generated and employed mice with epithelial cell-specific knock-in expression of the GSDMD N-terminal fragment ( *Gsdmd*-NT ^F/+^ *Villin*-CreERT) and epithelial cell-specific rescue expression of the GSDMD N-terminal fragment in a *Gsdmd*-deficient background ( *Gsdmd* ^−/−^ + *Gsdmd*-NT ^F/+^ *Villin*-CreERT). On the 9th day post-infection, compared with the WT mice, the *Gsdmd*-NT ^F/+^ *Villin*-CreERT and *Gsdmd* ^−/−^ + *Gsdmd*-NT ^F/+^ *Villin*-CreERT mice exhibited weakened *C. rodentium* signals ( ***Fig. 5A***). On the 15th day post-infection, these mice showed a reduced bacterial burden in the colon and mesenteric lymph nodes ( ***Fig. 5B***), lower colon tissue pathology scores ( ***Fig. 5C***), and decreased bacterial colonization ( ***Fig. 5D***). Collectively, these results suggest that the GSDMD in colon epithelial cells protects against intestinal *C. rodentium* infection through its N-terminal fragment.

## Discussion

Some evidence suggests that the inflammasome, an innate immune protein complex, is activated during intestinal pathogen infections and plays a critical role in the host's defense response and maintenance of intestinal immune homeostasis ^[ 30] ^. GSDMD, a key mediator of pyroptosis downstream of the inflammasome, significantly affects intestinal mucosal immunity and the onset of intestinal disease ^[ 31] ^. In addition to its well-documented role in cell death, GSDMD has recently been implicated in the processes that contribute to the integrity of the intestinal barrier. Zhang *et al* ^[ 24]^ discovered the involvement of GSDMD in cytoskeleton remodeling of goblet cells *via* actin regulation, which promotes mucous vesicle efflux under steady-state conditions. Our findings support the notion that the gasdermin protein does not affect the expression of intestinal mucosal proteins but is crucial for maintaining intestinal health. Recent investigations have revealed that *C. rodentium* may modulate the host F-actin to establish a foundation that aids its colonization and adhesion in the intestinal epithelium ^[ 32] ^. This raises the possibility that during the *C. rodentium* infection, GSDMD in intestinal epithelial cells may interfere with the formation of the host's actin base through its N-terminal functional fragment, potentially influencing bacterial infection and colonization. However, future investigation is warranted to elucidate the precise mechanisms involved.

Besides, our previous study added another layer of complexity to the role of GSDMD in intestinal immunity ^[ 25] ^, in which we found that GSDMD in intestinal macrophages regulated the onset of colitis and maintained intestinal immune homeostasis by modulating the cGAS-mediated inflammatory response, suggesting the importance of GSDMD in fine-tuning the immune response in the intestine and preventing excessive inflammation.

Therefore, GSDMD has a complex regulatory role in maintaining the intestinal mucosal immune barrier against pathogen infection and preserving homeostasis. Further elucidation is required to determine the specific intestinal cell type through which GSDMD exerts its defensive function.

The current study enhances our understanding of the role of GSDMD in intestinal immunity, revealing that the epithelial-specific GSDMD-deficient mice have the reduced defense against intestinal bacterial infection. Interestingly, this protective effect is mediated by the N-terminal segment of GSDMD, but is independent of its effect on antimicrobial peptide expression or mucin secretion. Thus, this suggests that GSDMD may have additional, hitherto unknown functions in non-immune cells that contribute to its protective effects against intestinal bacterial infection.

One potential mechanism by which the N-terminal segment of GSDMD may exert its protective effect is through promoting the release of inflammatory mediators, such as IL-1b and IL-18, by forming pyroptotic pores instead of cell death. These cytokines are known to play a crucial role in the host defense against infection and may be released through pores formed by the N-terminal oligomerization of GSDMD. Miao *et al* ^[ 33]^ highlighted that this segment might also damage mitochondria, leading to the release of these cytokines, suggesting a role for mitochondrial dysfunction in GSDMD-mediated infection protection. Alternatively, GSDMD may regulate the expression or activity of other unknown antimicrobial molecules. Future studies are warranted to explore these possibilities and to elucidate the precise molecular mechanism underlying the protective effect of GSDMD.

In conclusion, our work has unveiled a new dimension to the role of GSDMD in intestinal immunity. By protecting intestinal epithelial cells against bacterial infection through its N-terminal segment, GSDMD emerges as a critical player in maintaining intestinal health and preventing intestinal bacterial infections. The current study not only deepens our understanding of the formation and regulation of intestinal mucosal immunity but also may open new avenues for developing novel strategies to combat intestinal infections.
